# Age, Sex, and Central Adiposity as Determinants of Autonomic Nervous System Reactivity in Adults

**DOI:** 10.3390/medicina61091682

**Published:** 2025-09-17

**Authors:** Ivan Majerčák, Ivana Jochmanová, Miloš Šlepecký, Antónia Kotianová, Michal Kotian, Ján Praško, Marta Zaťková

**Affiliations:** 1 Center for Obesity Treatment, Medical Group Košice s.r.o. and Faculty of Medicine, Pavol Jozef Šafárik University in Košice, Mudroňova 29, 040 01 Košice, Slovakia; 2 Institute of Nuclear and Molecular Medicine and Department of Nuclear Medicine, Faculty of Medicine, Pavol Jozef Šafárik University in Košice, Rastislavova 43, 042 53 Košice, Slovakia; ivana.jochmanova@upjs.sk; 3 1st Department of Internal Medicine, Teaching Hospital of Louis Pasteur and Faculty of Medicine, Pavol Jozef Šafárik University in Košice, Tr. SNP 1, 040 01 Košice, Slovakia; 4 Faculty of Social Sciences and Health Care, Constantin the Philosopher University in Nitra, Kraskova 1, 949 74 Nitra, Slovakia; 5 ABC Slovak Institute for CBT Training, Garbiarska 3948/8, 031 01 Liptovský Mikuláš, Slovakia; 6 Department of Psychiatry, University Hospital Olomouc and Faculty of Medicine, Palacky University, 775 15 Olomouc, Czech Republic; 7 Jessenia Inc. Rehabilitation Hospital Beroun, CDR, Akeso Holding, MINDWALK, s.r.o., 266 56 Beroun, Czech Republic

**Keywords:** autonomic nervous system, maladaptive coping strategies, life stress burden, sex differences, age differences, quality of life, lay–stand–lay test

## Abstract

*Background and Objectives:* The autonomic nervous system (ANS) orchestrates adaptation to stress; however, its reactivity is influenced by demographic, anthropometric, and psychosocial factors. While arterial stiffness and central adiposity are established cardiovascular risk markers, less is known about how maladaptive coping strategies, cumulative life stress, and quality of life influence short-term autonomic regulation. This study examined the age- and sex-specific associations between anthropometry, maladaptive coping, life stress, quality of life, and ANS adaptation in adults. *Materials and Methods:* In this cross-sectional study, 122 healthy adults aged 21–78 years underwent a standardized lay–stand–lay (LSL) protocol with pulse wave analysis. Hemodynamic outcomes included pulse wave velocity (PWVao), augmentation indices (AIxA and AIxB), and aortic blood pressures (SBPao and PPao). Anthropometric measures comprised BMI, waist and hip circumference, waist-to-hip ratio (WHR), and waist-to-height ratio (WHtR). Psychosocial assessments included the Young Hypercompensation Inventory (maladaptive coping), Holmes–Rahe Life Events Inventory (life stress), and EQ-5D-3L (quality of life). Associations were analyzed using mixed-effects models adjusted for covariates, with false discovery rate correction. *Results:* Age was the strongest determinant of autonomic reactivity: older adults showed greater recovery of augmentation indices and central pressures after orthostatic challenge. Sex differences were evident, with women displaying consistently higher augmentation indices and men showing greater PWV responses. Central adiposity (WHR, WHtR, and waist circumference) predicted blunted augmentation index reactivity, while hip circumference was protective. BMI-defined obesity showed weaker associations. Maladaptive coping, life stress burden, and quality of life were not significantly associated with ANS indices after correction for multiple comparisons. *Conclusions:* ANS adaptation to postural stress is largely determined by age, sex, and visceral adiposity, whereas psychosocial measures showed limited influence in this healthy adult sample. These findings highlight the demographic and anthropometric determinants of cardiovascular adaptability, suggesting that psychosocial influences may primarily act through long-term behavioral and neuroendocrine pathways.

## 1. Introduction

Adaptation to stress is a fundamental function of the autonomic nervous system (ANS), which orchestrates physiological responses to maintain homeostasis in the face of internal and external demands. Chronic stress entails sustained activation of both parts of the ANS, i.e., the sympathetic and parasympathetic nervous systems, resulting in elevated cortisol and catecholamine levels that promote oxidative stress, endothelial dysfunction, and inflammation [1]. Persistent psychosocial stress can increase energy intake, preference for highly palatable foods, and sleep disruption, fostering weight gain; excess adiposity may in turn act as a stressor that exacerbates neuroendocrine dysregulation [2].

The dynamic flexibility of the ANS can be assessed through cardiovascular reactivity and recovery in response to positional changes, such as those in supine-to-standing transitions, which reflect baroreflex sensitivity and vascular tone regulation [3]. Measures such as pulse wave velocity (PWV), augmentation indices (AIxs), and aortic blood pressures (BPao) offer valuable insights into vascular stiffness and autonomic modulation [4,5]. However, physiological reactivity does not occur in isolation—it is closely intertwined with psychological and behavioral factors.

How individuals perceive and manage stress affects both psychological well-being and physiological regulation. Coping strategies are often categorized as problem-focused, emotion-focused, or meaning-focused. Problem-focused coping aims to modify the source of stress through active efforts or seeking instrumental support, whereas emotion-focused coping involves strategies such as distraction, avoidance, or emotional support to regulate the emotional impact of stress [6]. Meaning-focused coping draws on personal values and beliefs to reframe adverse experiences and has been linked to resilience [6].

Alongside these adaptive forms, maladaptive coping patterns play a crucial role in stress reactivity and long-term health. The Young Hypercompensation Inventory (YCI) operationalizes coping responses derived from early maladaptive schemas, such as excessive striving for achievement, denial of personal vulnerability, or rigid control over emotions [7]. These hypercompensatory strategies may initially serve to buffer stress but often perpetuate psychological strain and physiological dysregulation. Evidence suggests that maladaptive coping is associated with heightened autonomic arousal, reduced recovery following stressors, and unhealthy behaviors—including overeating, smoking, and physical inactivity—that further impair cardiovascular resilience.

Coping is influenced by sex and sociocultural context. Large surveys reveal that women typically report higher perceived stress and employ more emotion-focused strategies—such as self-distraction, acceptance, and seeking emotional support—than men [8]. In contrast, men are more likely to use problem-focused strategies, such as planning and active coping [8]. Women also experience more frequent stressful life events and are subject to hormonal and psychosocial influences that alter stress responses [9]. With age, perceived stress decreases in women but may increase in men [9], indicating that coping trajectories are both sex- and age-specific. Despite these documented differences, most research has examined coping and physiological reactivity separately; few studies have simultaneously considered psychological strategies, life event burden, and cardiovascular adaptability across demographic strata.

### 1.1. Anthropometric Measures and Their Health Relevance

Body size and fat distribution provide another important context for stress adaptation. Body mass index (BMI), defined as weight (kg) divided by height squared (m^2^), classifies adults as underweight (<18.5), healthy weight (18.5–<25), overweight (25–<30), or obese (≥30). Obesity is further subdivided into classes 1 (30–<35), 2 (35–<40), and 3 (≥40). Central obesity, typically assessed by waist-to-hip ratio (WHR) or waist-to-height ratio (WHtR), is a stronger predictor of cardiometabolic risk than BMI alone. Elevated WHR or WHtR reflects visceral fat accumulation, which is associated with impaired baroreflex function, arterial stiffness, and hypertension. PWV measures the speed at which the blood pressure waveform travels along an artery; it is calculated as the ratio between the distance the pulse travels and the time required to traverse that distance [10]. AIxs, an output of pulse wave analysis, quantify the contribution of reflected pressure waves to the aortic pressure curve and are expressed as the percentage ratio of augmentation pressure to pulse pressure; increases in AIxs indicate faster forward pulse propagation and earlier wave reflection [10]. Both PWV and AIxs increase with age and central adiposity, and they predict cardiovascular events. Stress and body composition are interrelated: population studies show that cumulative psychosocial stressors across the life course increase the odds of overweight and obesity, while weight stigma and depressive symptoms can lead to maladaptive eating and further weight gain [2]. However, it remains unclear whether anthropometric indices themselves influence stress perception or coping behaviors.

### 1.2. Integrating Psychological, Anthropometric, and Autonomic Perspectives

The ANS orchestrates cardiovascular adaptation to positional changes via the baroreflex, which adjusts heart rate and vascular tone. When transitioning from supine to standing, blood pressure briefly falls before baroreceptors trigger sympathetic activation; upon returning to supine, parasympathetic activity restores homeostasis. Metrics such as PWV, AIxs, and BPao measured during lay–stand–lay (LSL) tests provide insights into autonomic flexibility and vascular stiffness. Most studies evaluating PWV and AIxs focus on disease risk, pharmacological interventions, or aging; few have related these physiological measures to psychological coping and life stress. Furthermore, evidence for sex- and age-specific interactions between body composition, coping strategies, and autonomic adaptation is scarce.

Importantly, not all coping mechanisms are adaptive. Hypercompensatory patterns, as described by Young, represent maladaptive strategies that may intensify autonomic load and impair recovery following stress. By considering these patterns alongside anthropometric and physiological markers, it becomes possible to clarify whether maladaptive coping constitutes an additional pathway linking psychosocial stress to cardiovascular dysregulation.

The present study addresses these gaps by examining how maladaptive stress-coping strategies, cumulative life stressors, and quality of life relate to ANS adaptability in response to postural changes and whether these associations vary by age, sex, and anthropometric characteristics. We hypothesized that (a) age and sex would be major determinants of autonomic reactivity and recovery; (b) central adiposity would be associated with impaired vascular adaptation; and (c) psychosocial measures (maladaptive coping, life events burden, and quality of life) would also be associated with abnormal ANS reactivity. To capture maladaptive coping patterns rooted in early maladaptive schemas, we used the YCI [7], which identifies hypercompensatory strategies that may perpetuate stress and hinder physiological recovery. The Holmes–Rahe Life Events Inventory (LEI) [11] was employed to quantify recent life stressors, while the EQ-5D-3L instrument assessed health-related quality of life. Anthropometric measures were obtained using standard protocols, and cardiovascular reactivity was indexed by PWV, AIxs, and aortic pressure changes during an LSL test.

By integrating psychometric, anthropometric, and physiological data in a population-based sample, we aim to elucidate the biopsychosocial determinants of stress adaptation and highlight potential targets for interventions.

## 2. Subjects and Methods

### 2.1. Study Design and Participants

This quantitative, non-interventional, and observational study examined ANS reactivity during an LSL test. Participants for this cross-sectional observational study were recruited from the general population. The inclusion criteria required participants to be free of acute or chronic illnesses that could affect ANS functioning. Individuals with cardiovascular disease, with diagnosed psychiatric disorders, or on medications influencing cardiovascular or nervous system regulation were excluded. Participants were stratified by sex, age group, and partnership status.

### 2.2. Instruments and Measures

Questionnaires: Data on partnership, physical activity, and smoking status were collected via structured interviews or questionnaires. Stress exposure was quantified with the Holmes–Rahe LEI [11]. At the same time, maladaptive coping strategies were evaluated using the YCI, which includes subscales for individuality (YCI-I), personal control (YCI-PC), and social control (YCI-SC). Health-related quality of life was assessed using the EQ-5D-3L questionnaire.

Anthropometry and vital signs: Anthropometric parameters, including sex, age, height, weight, waist, and hip circumference, were measured according to standard protocols. BMI, WHR, and WHtR were calculated accordingly. Systolic and diastolic blood pressure (SBP and DBP, respectively) and heart rate (HR) were measured twice and averaged.

LSL test: Autonomic reactivity and adaptation were evaluated using a standardized LSL test: 5 min supine (L1), 2 min standing (S), and 5 min supine (L2). Hemodynamic parameters were measured using an Arteriograph device (Tensiomed, Budapest, Hungary); two readings per phase were averaged for HR, respiratory rate (RR), systolic BPao (SBPao), aortic pulse wave velocity (PWVao), aortic (AIxA) and brachial AIxs (AIxB), and aortic pulse pressure (PPao). Reactivity indices (Δ) were computed as phase differences (e.g., ΔHR = HR_S − HR_L1; recovery = HR_S − HR_L2).

### 2.3. Statistical Analysis

All analyses were performed in JASP v.0.19.3.0 (JASP Team, University of Amsterdam, Amsterdam, The Netherlands). Normality was assessed using Shapiro–Wilk tests, histograms, and Q–Q plots. Because many variables were non-normally distributed, we used nonparametric tests and robust regression. Continuous data are summarized as median (range) and categorical data as n (%). Between-sex comparisons used Wilcoxon–Mann–Whitney tests with Hodges–Lehmann median difference estimates and χ^2^/Fisher’s exact tests for categorical variables. *p*-values were not multiplicity-adjusted for descriptive values (L1, S, L2, and their deltas).

Autonomic reactivity during the LSL test was expressed as ΔL1S = S − L1, ΔSL2 = L2 − S, and ΔL1L2 = L2 − L1 for AIxA, AIxB, SBPao, PPao, PWVao, and HR.

Confirmatory inference used two complementary approaches: 1) Phase-based linear mixed-effects models (LMMs): random intercepts for participants and fixed effects for the phase and predictor with orthogonal contrasts (c1: L1→S reactivity; c2: S→L2 recovery) and phase × predictor interactions. 2) Δ-based models (primary): ordinary least squares (OLS)/analysis of covariance (ANCOVA) with heteroskedasticity-consistent standard errors (HC3). Each Δ was modeled as an outcome, adjusting for age (z), sex, smoking, partnership, and the relevant baseline (ΔL1S|L1, ΔSL2|S, and ΔL1L2|L1). Continuous predictors were z-scored; results are reported as standardized coefficients (β_std).

Obesity was analyzed both categorically (overall obesity, high waist, high WHR, and WHtR ≥ 0.5) and continuously (BMI, waist, hips, WHR, and WHtR). When multiple anthropometric variables were entered into a model, multicollinearity was checked, and predictors with a variance inflation factor (VIF) > 10 were removed.

Associations between psychological measures (LEI, YCI subscales, and EQ-5D-3L) and autonomic Δs were tested with standardized OLS models (z-Δ ~ z-predictor + covariates; HC3). As context, we computed Spearman correlations overall and by sex. Sex specificity was examined using both sex-stratified Δ-models and pooled interaction models (z-Δ ~ z-predictor + sex + predictor × sex + covariates). Differences between sexes were interpreted only when the predictor × sex term was significant, consistent with best practices in interaction testing [12].

Multiplicity was controlled with the Benjamini–Hochberg false discovery rate (FDR) correction within pre-specified families: within outcome for LMM interactions and Δ-model grids and within outcome for psychological analyses and interaction *p*-values. For analyses where JASP provides built-in options (correlation matrices and pairwise tests), FDR was applied directly. For regression and mixed-effects models, *p*-values were exported and adjusted in R (R Core Team, Vienna, Austria) using the p.adjust function (method = “BH”). We report β_std with 95% confidence intervals, coefficient of determination (R^2^), number of complete cases (n), and q (FDR-adjusted). Significance was defined as q < 0.05.

## 3. Results

### 3.1. Demographic Data and Measurements

The present study included 122 adult participants (32.79% males and 67.21% females) aged between 21 and 78 years, with a median age of 40.5 years. Demographic data and anthropometric parameters are shown in Table 1. Significant differences between males and females were noted in all anthropometric parameters and general hemodynamic parameters, except HR. There were no differences in the psychological and coping measures between males and females (Table 1). Measurements of parameters taken during the LSL test and calculated deltas are listed in Table 2. Statistically significant differences between sexes were found in ΔRR between the L1 and L2 phases and in ΔPWVao L1-S and S-L2 of the LSL test.

### 3.2. Hemodynamic Parameters During the LSL Test

During the LSL test, HR, RR, AIxA, AIxB, SBPao, PPao, and PWVao were measured in L1, S, and L2, and Δ values were calculated. HR and RR rose on standing and fell on returning to supine, as expected; no sex differences were detected for any phase or Δ values, except an RR change from L1 to L2, which was greater in women (*p* = 0.003). Women showed higher AIxs across all phases—AIxA: L1 (*p* = 0.018), S (*p* = 0.006), and L2 (*p* = 0.010); AIxB: L1 (*p* = 0.017), S (*p* = 0.007), and L2 (*p* = 0.022)—while ΔAIxA and ΔAIxB were nonsignificant (all *p* > 0.05) (Table 2). SBPao and PPao showed no sex differences in absolute values or Δs (all *p* > 0.05). PWVao increased more in men during standing (*p* = 0.002), with higher ΔL1–S (*p* = 0.014) and ΔS–L2 (*p* = 0.012); other phase comparisons were nonsignificant. Thus, men exhibited more pronounced PWVao reactivity to orthostatic load and return to supine. This RR difference is from the descriptive table (unadjusted for multiple comparisons); confirmatory models apply multiplicity correction elsewhere in the manuscript.

### 3.3. Influence of Demographic and Lifestyle Factors on ANS Reactivity

We examined whether age, sex, partnership status, smoking, and physical activity predicted autonomic reactivity (Δ values between L1, S, and L2) in the LSL test (Supplementary Table S1). We evaluated predictors using two complementary frameworks: (1) linear mixed-effects models with orthogonal contrasts (c1: L1→S; c2: S→L2) to test phase × predictor interactions and (2) Δ-models (ANCOVA with HC3; baselines ΔL1S|L1, ΔSL2|S, and ΔL1L2|L1) to confirm phase-to-phase changes. Multiple testing was controlled with Benjamini–Hochberg FDR within pre-specified families: for Δ-models, separately within each (Outcome × Delta) (ΔL1S, ΔSL2, ΔL1L2), and for mixed models, within each (Outcome) across the set of phase × predictor interactions.

Across frameworks, age showed the most consistent FDR-significant associations. Older participants displayed stronger recovery (ΔSL2) in wave reflection indices (AIxA, AIxB), central pressures (SBPao and PPao), and aortic stiffness (PWVao). In the mixed models, age was also FDR-significantly related to greater standing-related decreases (c1) in AIxA/AIxB/SBPao/PPao and a smaller standing rise in PWVao, indicating a coherent age pattern across both reactivity and recovery. A modest net change (ΔL1L2) in AIxA with age also survived FDR.

Sex effects were limited to aortic stiffness: men showed greater PWVao reactivity, significant in both the Δ-models (ΔL1S) and the mixed-model reactivity contrast (c1). Smoking was associated with a blunted recovery of augmentation indices (ΔSL2) and greater standing reactivity (c1) for wave reflection and central pressure. Partnership did not yield FDR-significant effects in either framework.

No FDR-significant associations were observed for HR or RR reactivity or recovery; an isolated RR net change finding is detailed in the tables. Full effect sizes (β per 1 SD, where applicable), 95% CIs, and q-values are provided in the accompanying result tables; the text highlights only those findings that remained significant within their respective FDR families.

### 3.4. Anthropometric Factors and ANS Reactivity

After Benjamini–Hochberg FDR control within each (Outcome × Δ) family, indices of central adiposity were consistently related to reactivity (Supplementary Table S2). On standing (ΔL1S), higher BMI, WHR, WHtR, and waist were associated with larger increases in AIxA and AIxB. The clearest example was WHR → AIxB (q < 0.01). Waist and WHtR also predicted higher SBPao on standing and higher PPao. In contrast, greater hip circumference was related to lower standing reactivity for PPao and PWVao despite a concurrent increase in AIxB (all q < 0.05).

During recovery (ΔSL2), adiposity measures predicted larger decreases across several outcomes. Hip circumference showed the strongest recovery effects across multiple outcomes (e.g., AIxA with q < 0.001). BMI, WHR, WHtR, and waist circumference also tracked greater AIxA decreases in recovery (all q ≤ 0.022) and AIxB decreases (q ≤ 0.026). For net change (ΔL1L2), hip circumference remained inversely associated with AIxA (q = 0.032) and PPao (q = 0.0033).

No anthropometric phase-interaction effects survived FDR in the LMM (c1/c2) framework; all FDR-significant findings arose from the Δ-model ANCOVA (HC3) analyses, with β interpreted per 1 SD in each anthropometric predictor.

### 3.5. Effect of Obesity and Central Adiposity

Comparisons of obese vs. non-obese participants (Supplementary Table S3) revealed no significant differences in most Δ parameters (HR, RR, AIxA/B, SBPao, and PPao). The notable exception was PWVao: obese individuals showed a lower fall on standing and a larger rise on return to supine, with ΔPWVao_SL2 significantly higher (*p* < 0.05), suggesting reduced vascular recovery. In adjusted Δ-models (covariates: age-z, sex, smoking, and partnership) with FDR control within each outcome, categorical obesity indicators (overall obesity, high waist, high WHR, and WHtR ≥ 0.5) showed no associations with postural reactivity that survived correction (Supplementary Table S4). In contrast, continuous central adiposity (per 1-SD higher WHR, WHtR, or waist circumference) was consistently related to attenuated wave reflection responses: for AIxA, higher adiposity predicted a smaller fall on standing (ΔL1S, q ≈ 0.016–0.018) and a smaller rebound on return to supine (ΔSL2, q ≈ 0.016–0.018). AIxB showed the same pattern with larger magnitudes (ΔL1S, ΔSL2, q ≈ 0.013, Supplementary Table S4). Model fit for these associations was moderate (R^2^ ≈ 0.18–0.25). No FDR-significant effects of central adiposity were observed for PPao, SBPao, PWVao, or HR deltas. In sex-stratified analyses, these central adiposity effects were present in women (multiple AIxA/AIxB tests q < 0.05) but did not survive FDR in men, reinforcing that higher central adiposity is associated with blunted augmentation index reactivity to posture, without detectable effects on pressure, stiffness, or HR deltas after multiple-testing adjustment.

### 3.6. Relationship Between Psychological and Anthropometric Factors

Age was the only anthropometric measure to show meaningful links with maladaptive coping. It correlated negatively with all three YCI subscales—individuality (r_s_ = −0.32, q < 0.01), social control (r_s_ = −0.25, q < 0.05), and YCI-AVG (r_s_ = −0.29, q < 0.01). Correlations with YCI-PC were small and not robust after FDR, and associations with LEI and EQ-5D-3L were negligible. No other anthropometric variables (weight, height, BMI, waist or hip circumferences, WHR, and WHtR) were significantly correlated with maladaptive stress-coping, LEI, or EQ-5D-3L scores after FDR; all correlations were small and nonsignificant, suggesting body composition does not play a direct role in these psychological measures (Table 3).

In sex-stratified Spearman analyses, more nominal signals appeared in women (e.g., age correlated with YCI-SC and YCI-I; height inversely correlated with YCI-PC; some positive correlations of central size with YCI-PC), while men showed fewer associations (e.g., age with YCI-I and WHR with LEI). However, these descriptive differences were formally evaluated with interaction tests in the multivariable framework (see below).

In pooled models adjusting for smoking and partnership, age was independently and inversely associated with YCI-I (β_std ≈ −0.35, 95% CI roughly −0.57 to −0.12; q ≈ 0.015). For YCI-PC, both greater waist circumference (β_std ≈ +0.70, ~0.13 to 1.26; q ≈ 0.030) and older age (β_std ≈ −0.27, ~−0.49 to −0.05; q ≈ 0.030) were associated with scores, while male sex had a lower adjusted YCI-PC (β_std ≈ −0.87, ~−1.45 to −0.29; q ≈ 0.019). Model fit was modest (R^2^ ~ 0.10–0.12). No robust multivariable associations emerged for LEI, EQ-5D-3L, or the remaining YCI indices after FDR.

When models were stratified by sex, several predictors were FDR-significant in women (e.g., age with YCI totals/average/subscales and age/waist/hips with YCI-PC), whereas few reached FDR in men. Critically, formal sex × predictor interaction tests (pooled models: outcome ~ sex + predictor + sex × predictor + covariates, with FDR within outcome) were not significant (Table 4). Thus, although more associations met significance thresholds in women, we cannot conclude that effects differ between women and men; the apparent disparity is compatible with power/sample size and variability differences rather than true sex-specific effects.

### 3.7. Relationship Between Life Stress, Maladaptive Coping Strategies, and Autonomic Regulation

We tested whether life stress (Holmes–Rahe LEI), maladaptive coping strategies (YCI subscales: YCI-I, YCI-SC, YCI-PC, and YCI-AVG), and quality of life (EQ-5D-3L) were associated with autonomic regulation during the LSL test. Primary analyses used standardized OLS (z-outcome and z-predictor), adjusted for age, smoking, and partnership; models were fit separately for each outcome × delta × psychological predictor combination, with Benjamini–Hochberg FDR correction within each physiological outcome. Model fit was summarized with R^2^.

Across all outcomes and deltas, no associations survived FDR correction (all q ≥ 0.05, Supplementary Table S5). Point estimates were generally small, and R^2^ values were modest, indicating that variation in LEI, maladaptive coping (YCI subscales/average), or EQ-5D-3L did not explain a meaningful amount of variance in the autonomic deltas after adjustment and multiple-testing control. Sensitivity analyses that additionally adjusted for BMI (z) or waist circumference (z) produced the same conclusion (no FDR-significant findings).

### 3.8. Sex-Specific Findings

We repeated the standardized OLS models stratified by sex (females and males analyzed separately with the same covariates) and applied FDR within the outcome in each stratum. As in the pooled analyses, no associations met FDR significance in either sex across base or sensitivity models. To formally assess whether apparent differences between females and males could reflect true effect modification, we fit pooled interaction models (z-Δ ~ z-predictor + sex + predictor × sex + covariates) and applied outcome-wise FDR to the sex × predictor terms. No sex × predictor interactions survived FDR (all q_interaction ≥ 0.05). Thus, while some nominal (uncorrected) effects appeared more frequently in one sex in descriptive tables/plots, these did not constitute reliable sex differences once tested formally and corrected for multiplicity.

In summary, within this cohort, indices of life stress, maladaptive coping style, and quality of life were not robustly associated with autonomic reactivity to posture change, and we found no evidence for sex-specific effects after multiple-testing control.

## 4. Discussion

The present study examined the associations between anthropometric characteristics, maladaptive stress-coping strategies, life stress burden, quality of life, and ANS reactivity in a community-based adult cohort using a standardized LSL test. Our main findings can be summarized as follows: (1) age emerged as the strongest determinant of ANS reactivity, with older adults showing more pronounced recovery of wave reflection, central pressures, and arterial stiffness indices; (2) sex differences were evident in vascular responses, with women showing higher AIxs (A, B) across all phases and men demonstrating greater PWV reactivity to orthostatic stress; (3) indices of central adiposity—including WHR, WHtR, and waist circumference—were consistently associated with blunted AIx reactivity, whereas hip circumference was protective; and (4) contrary to expectations, maladaptive coping strategies, as assessed by the YCI, recent life stressors (LEI), and self-reported quality of life (EQ-5D-3L), were not significantly linked to ANS reactivity after multiple-testing correction. Together, these findings underscore the pivotal roles of age, sex, and visceral adiposity in shaping vascular and autonomic responses to stress. In contrast, psychosocial variables may exert subtler or context-dependent effects.

### 4.1. Age-Related Differences in Autonomic Reactivity

Aging is accompanied by structural and functional changes in the cardiovascular system, including arterial stiffening, reduced elasticity, and impaired baroreflex sensitivity [5,13,14]. In the present study, older participants displayed stronger recovery (ΔSL2) in AIxA, AIxB, central pressures (SBPao and PPao), and PWVao following the orthostatic challenge. Interestingly, mixed-effects models also showed that older adults exhibited greater standing-related decreases in AIxs and smaller rises in PWVao, suggesting altered hemodynamic strategies during orthostatic adaptation. While these counterintuitive findings may reflect compensatory vascular responses, they might represent maladaptive overcompensation that preserves short-term homeostasis at the expense of vascular burden over time [15,16].

These findings align with prior work, which shows that arterial stiffness rises progressively with age and predicts cardiovascular events [5,13], while baroreflex sensitivity declines [14]. Our results extend these observations by showing that older individuals may nevertheless engage compensatory vascular mechanisms that produce greater rebound in AIxs during recovery. This pattern may reflect increased reliance on peripheral vasoconstriction and altered wave reflection sites in aging arteries [15].

From a clinical perspective, these findings reinforce that autonomic and vascular adaptation to stress is not static but shifts across the life course. The paradoxical “stronger recovery” observed in older adults may represent maladaptive overcompensation, which, while preserving blood pressure stability acutely, contributes to chronic vascular load and left ventricular hypertrophy [16].

### 4.2. Sex Differences in Autonomic Function

We found that women consistently had higher AIxs than men in all LSL phases, whereas men exhibited stronger PWV responses to standing and returning to the supine position. These findings are consistent with known sex-related vascular differences. Women generally exhibit higher AIxs values at younger ages, which is attributed to a shorter stature, smaller arterial caliber, and greater wave reflection from peripheral sites [17]. DuPont et al. [18] further demonstrated that estrogen deficiency after menopause accelerates vascular stiffening, thereby narrowing sex differences with aging.

In contrast, higher PWV responses in men are compatible with their generally greater baseline arterial stiffness and more pronounced sympathetic vasoconstriction [19]. Hay [20] reported that men demonstrate stronger sympathetic responses to orthostatic challenges, while women show greater vagal modulation. Together, these data suggest that women may be more vulnerable to increased wave reflection, while men experience larger dynamic changes in arterial stiffness. These patterns underscore the need for sex-specific normative values when interpreting PWV and AIxs in both research and clinical contexts.

### 4.3. Central Adiposity and Vascular Adaptability

One of the most robust findings of our study is that central adiposity, but not categorical obesity, was consistently associated with impaired vascular reactivity. WHR, WHtR, and waist circumference predicted blunted falls and rebounds in AIxs during postural change, while hip circumference showed protective associations.

These results are consistent with meta-analyses and cohort studies emphasizing that visceral adiposity, rather than BMI, drives cardiometabolic risk. Czernichow et al. [21] demonstrated that WHR and waist circumference outperform BMI in predicting cardiovascular mortality. Lear et al. [22] showed ethnic variation in body fat distribution but consistently found central adiposity to be the critical determinant of metabolic risk. Emamat et al. [23] confirmed, in a systematic review, that abdominal obesity strongly predicts cardiovascular mortality, independent of BMI.

Mechanistically, visceral adiposity is linked to endothelial dysfunction, impaired nitric oxide bioavailability, and chronic inflammation [24]. Autonomic dysfunction in obesity is characterized by increased sympathetic tone and reduced baroreflex sensitivity [25]. Our finding that hip circumference is inversely correlated with AIxs changes supports the notion that gluteofemoral fat is metabolically protective, as previously described by Manolopoulos et al. [26]. Thus, fat distribution, not overall adiposity, appears to be the key determinant of vascular adaptability.

### 4.4. Maladaptive Coping, Life Stress, and Self-Reported Quality of Life

Contrary to expectations, maladaptive coping patterns (YCI subscales), cumulative life stress (LEI), and quality of life (EQ-5D-3L) were not significantly associated with autonomic indices after adjustment and FDR correction. Previous studies have reported links between maladaptive coping and cardiovascular dysregulation, including heightened sympathetic activation, blunted HR variability, and increased blood pressure reactivity [27,28,29,30]. Our null findings may reflect the relatively healthy community-based sample, which excluded individuals with manifest psychiatric or cardiovascular disease, thereby reducing variability in coping pathology. Moreover, cross-sectional design and self-report psychometrics may underestimate the dynamic relationship between stress responses and physiological reactivity.

Life events burden (LEI). The cumulative number of stressful life events has been consistently linked to cardiovascular morbidity and mortality in prospective studies. The original Holmes–Rahe LEI demonstrated dose–response relationships between event burden and risk of illness [11]. More recent cohort studies confirm that exposure to multiple stressful life events predicts the development of incident hypertension, metabolic syndrome, and increased arterial stiffness [31,32]. In our cohort, however, LEI scores were not associated with ANS reactivity. A likely explanation is the relatively modest burden of severe events in this community-based sample. Moreover, it is plausible that life events exert their influence through chronic pathways—via endocrine dysregulation, inflammation, and behavioral risk factors—rather than through acute hemodynamic responses to postural change.

Self-reported quality of life (EQ-5D-3L). Poor self-reported quality of life has been linked to increased cardiovascular risk and mortality [33,34]. For example, lower EQ-5D scores are associated with greater all-cause and cardiovascular mortality in population studies, even after adjustment for comorbidities [35]. The lack of association in our study may again reflect the relatively healthy profile of participants, with restricted variance in EQ-5D-3L responses. Furthermore, quality of life is a broad construct, influenced by physical health, mental well-being, and social context; its effects on ANS reactivity may be indirect, mediated through health behaviors and chronic disease, rather than directly observable in short-term physiological testing.

Developmental perspective. We did observe age-related decreases in maladaptive coping scores, consistent with longitudinal evidence that emotional regulation improves with age [36,37]. Charles et al. [38] showed that older adults exhibit reduced affective reactivity to daily stressors, reflecting adaptive shifts in appraisal and coping. Thus, while maladaptive coping, life events, and quality of life may not have had a strong influence on ANS responses in our cohort, developmental trends remain evident and may impact long-term cardiovascular risk trajectories.

Taken together, these findings suggest that while psychosocial variables are important determinants of long-term health, their direct links to acute autonomic reactivity may be less pronounced in relatively healthy adults without significant comorbidity. Future studies should investigate these associations in clinical or high-risk populations, where variance in psychosocial stressors and quality of life is greater and may exert stronger physiological effects.

### 4.5. Integration and Implications

Taken together, our findings emphasize that short-term autonomic adaptability is driven predominantly by age, sex, and central adiposity. At the same time, psychosocial influences may exert their effects indirectly and over longer time horizons. Clinically, interventions targeting visceral fat reduction and the development of age- and sex-specific normative values for AIxs and PWV may improve the assessment of cardiovascular resilience.

### 4.6. Strengths and Limitations

The present study has several strengths. We integrated psychometric, anthropometric, and physiological assessments in a single population-based design, applied a standardized LSL test to probe autonomic adaptation, and used rigorous statistical models with correction for multiple testing. To our knowledge, few studies have simultaneously examined maladaptive coping, central adiposity, life event burden, quality of life, and ANS reactivity in a general adult sample.

However, the limitations must be acknowledged. The cross-sectional design precludes causal inference and cannot address whether maladaptive coping or life stress leads to physiological dysregulation over time. Several associations explained only a small proportion of variance (R^2^ ≤ 0.05). These findings should therefore be considered exploratory and interpreted with caution, reflecting the multifactorial nature of autonomic regulation and the relatively healthy composition of our sample. The sample size, although adequate for primary analyses, had limited power to detect subtle sex-specific effects, which may explain the lack of robust psychological associations. Psychometric measures, which rely on self-report, may be subject to recall and social desirability biases. Furthermore, while the YCI captures hypercompensatory coping, it does not encompass all maladaptive strategies, such as rumination or hostility, which may have stronger autonomic correlates. It also does not capture stress or other stress-coping styles. Finally, our partici-pants were generally healthy; therefore, the findings may not be generalized to clinical populations with cardiovascular or psychiatric diseases.

## 5. Conclusions

Age, sex, and central adiposity are the primary determinants of ANS adaptation to postural stress, whereas maladaptive coping, life events, and self-reported quality of life showed limited associations in this healthy adult sample. These findings highlight the significant role of demographic and body composition factors in shaping cardiovascular adaptability while suggesting that psychosocial influences primarily act through long-term behavioral and neuroendocrine pathways. Future longitudinal and interventional studies should investigate how coping, stress burden, and quality of life interact with central adiposity and aging to influence cardiovascular outcomes.

## Figures and Tables

**Table 1:** Demographic and anthropometric characteristics of the sample and selected measurements.

Subjects	Total	Males	Females	*p*
(N,%)	122	40 (32.79%)	82 (67.21%)	<0.001
Variable	Mean ± SD/n (%)			
Demographic and Lifestyle				
Age (years)	40.5 (27–78)	43.50 (21–78)	40 (21–70)	0.71
In Partnership (N,%)	80/122 (65.57%)	54/82 (65.85%)	26/40 (65.00%)	0.75
Smoking (yes; N,%)	27/122 (22.13%)	19/82 (23.17%)	8/40 (20.00%)	0.87
Physical Activity (hours per week)	4.0 (0.0–46.0)	4.0 (0.0–46.0)	3.0 (0–45.0)	0.21
Anthropometric Measurements				
Height (cm)	170.0 (156.0–196.0)	180.00 (169.00–195.00)	168.00 (156.00–180.00)	<0.001
Weight (kg)	67.36 (43.3–130.77)	85.06 (53.47–130.77)	62.89 (43.30–109.53)	<0.001
Waist Circumference (cm)	80.0 (59.0–130.0)	96.00 (76.00–130.00)	73.00 (59.00–111.00)	<0.001
Hip Circumference (cm)	99.0 (80.0–136.0)	103.00 (89.00–120.00)	96.00 (80.00–136.00)	<0.001
BMI (kg/m^2^)	23.35 (16.5–41.3)	26.30 (18.50–41.30)	22.20 (16.50–37.90)	<0.001
WHtR	0.47 (0.36–0.69)	0.52 (0.44–0.69)	0.44 (0.36–0.67)	<0.001
WHR	0.87 (0.71–1.23)	0.91 (0.78–1.07)	0.85 (0.71–1.23)	<0.001
Psychological and Coping Measures				
EQ-5D-3L	80 (30–100)	80 (30–100)	80.00 (40.00–100.00)	0.35
LEI (LCU)	108.5 (0–633.0)	102.00 (0.00–332.00)	109.00 (0.00–633.00)	0.77
YCI Total Score	140.5 (3.04–212.0)	146.00 (48.00–212.00)	139.00 (69.00–190.00)	0.53
YCI Average Score	2.93 (1.0–4.42)	3.04 (1.00–4.42)	2.90 (1.44–3.96)	0.41
YCI Social Control	2.71 (1.0–4.26)	2.84 (1.00–4.26)	2.63 (1.26–4.05)	0.30
YCI Individuality	3.30 (1.0–4.9)	3.40 (1.00–4.60)	3.20 (1.70–4.90)	0.26
YCI Personal Control	3.50 (1.0–5.5)	3.25 (1.00–5.50)	3.50 (1.00–5.50)	0.24
Hemodynamic parameters				
Systolic BP (mmHg)	123.5 (91.00–156.50)	132.50 (105.00–156.50)	118.50 (91.00–149.50)	<0.001
Diastolic BP (mmHg)	70.00 (48.50–94.50)	75.50 (56.00–94.50)	67.75 (48.50–149.50)	<0.001
HR (bpm)	71 (45.00–103.00)	70.00 (45.00–88.00)	71.50 (54.00–103.00)	0.29

All values are presented as the median (range) for continuous variables and as N (%) for categorical variables. Abbreviations: BMI—body mass index; BP—blood pressure; bpm—beats per minute; EQ-5D-3L—health-related quality-of-life questionnaire—quality-of-life score; HR—heart rate; LCU—Life Change Units; LEI—Holmes–Rahe Life Events Inventory Score; N—number; WHR—waist-to-hip ratio; WHtR—waist-to-height ratio; and YCI—Young Compensation Inventory.

**Table 2:** Hemodynamic parameters (LSL test).

Parameter	Phase	Total	Males	Females	*p*	HL (M-F)
HR (bpm)	L1	64.9 (38.78–94.19)	63.85 (38.78–92.04)	65.80 (45.57–94.19)	0.31	−2.16
	S	79.96 (47.19–114.05)	80.47 (47.19–108.17)	79.79 (55.27–114.05)	0.67	−0.95
	L2	64.16 (39.57–91.73)	64.16 (39.57–90.11)	64.44 (46.86–91.73)	0.36	−1.76
ΔHR (bpm)	L1-S	15.79 (1.05–41.13)	15.81 (8.09–41.13)	15.79 (1.05–35.70)	0.88	0.17
	S-L2	−17.01 (−39.44–−2.36)	−16.47 (−39.44–−6.63)	−17.23 (−38.10–−2.36)	0.94	−0.04
	L1-L2	−0.86 (−11.65–6.54)	−0.73 (−8.37–6.54)	−0.99 (−11.65–4.11)	0.66	0.20
RR (brpm)	L1	15.54 (6.48–18.92)	15.85 (8.30–18.65)	15.36 (6.48–18.92)	0.21	0.59
	S	14.67 (6.83–18.88)	14.64 (10.30–18.88)	14.68 (6.83–18.65)	0.55	0.26
	L2	15.69 (6.76–19.38)	15.91 (7.06–18.72)	15.63 (6.76–19.38)	0.10	0.00
ΔRR (brpm)	L1-S	−0.28 (−4.98–3.37)	−0.79 (−4.98–2.97)	−0.07 (−4.95–3.37)	0.66	−0.15
	S-L2	0.41 (−4.21–5.68)	0.25 (−4.21–4.32)	0.45 (−3.12–5.68)	0.21	−0.48
	L1-L2	0.22 (−4.15–3.58)	0.01 (−4315–2.16)	0.35 (−2.97–3.58)	0.003	−0.54
AIxA (%)	L1	25.0 (1.05–61.5)	19.50 (1.05–48.65)	27.83 (3.45–61.50)	0.02	−6.60
	S	16.28 (−3.1–54.95)	13.83 (−3.10–40.05)	21.05 (−0.30–54.95)	0.01	−6.50
	L2	27.55 (3.2–62.7)	22.03 (3.20–52.20)	29.45 (5.50–62.70)	0.01	−7.30
ΔAIxA (%)	L1-S	−6.3 (−33.5–17.45)	−6.10 (−31.20–17.45)	−6.63 (−33.5–11.20)	0.76	−0.65
	S-L2	8.35 (−16.75–38.7)	9.25 (−16.75–32.90)	8.08 (−8.80–38.70)	0.82	0.30
	L1-L2	2.05 (−8.3–15.2)	1.40 (−8.30–9.20)	2.33 (−5.58–15.20)	0.50	−0.40
AIxB (%)	L1	−24.93 (−72.35–47.15)	−35.90 (−72.35–21.80)	−19.35 (−67.50–47.15)	0.02	−13.00
	S	−42.25 (−80.5–34.2)	−47.08 (−80.50–4.70)	−33.25 (−74.90–34.20)	0.01	−12.50
	L2	−19.55 (−68.05–49.55)	−27.75 (−68.05–28.75)	−16.20 (−63.45–49.55)	0.02	−12.50
ΔAIxB (%)	L1-S	−12.4 (−66.15–34.5)	−12.00 (−31.50–34.50)	−13.13 (−66.15–20.10)	0.76	−1.55
	S-L2	17.08 (−33.1–76.45)	20.00 (−33.10–75.00)	15.95 (−17.35–76.45)	0.56	1.90
	L1-L2	4.03 (−16.4–76.75)	3.48 (−16.40–76.75)	4.48 (−9.85–30.05)	0.69	−0.53
SBPao (mmHg)	L1	122.18 (92.6–180.1)	124.15 (95.85–165.35)	119.40 (92.60–180.10)	0.44	2.50
	S	123.93 (101.8–187.8)	122.90 (102.15–187.80)	125.23 (101.80–158.70)	0.72	−1.10
	L2	121.90 (−2.0–170.15)	124.83 (−2.00–164.35)	121.48 (90.35–170.15)	0.66	2.15
ΔSBPao (mmHg)	L1-S	4.30 (−37.55–36.45)	4.00 (−32.15–36.45)	4.58 (−37.55–20.40)	0.44	−1.10
	S-L2	−3.35 (−117.87–34.75)	−3.40 (−117.85–34.75)	−3.15 (−20.50–27.60)	0.96	0.48
	L1-L2	0.85 (−119.5–17.95)	1.35 (−119.50–11.15)	0.50 (−11.80–17.95)	0.83	0.00
PPao (mmHg)	L1	44.78 (30.0–87.95)	43.75 (30.00–67.45)	46.28 (32.60–87.95)	0.28	−1.80
	S	39.93 (28.25–73.2)	40.68 (28.60–55.55)	39.78 (28.25–73.20)	0.48	−1.10
	L2	45.58 (−12.0–87.85)	45.05 (−12.00–69.25)	45.63 (32.50–87.85)	0.23	−1.55
ΔPPao (mmHg)	L1-S	−4.78 (−36.5–10.75)	−4.90 (−25.45–7.85)	−4.53 (−36.50–10.75)	1.00	0.00
	S-L2	5.38 (−49.85–27.1)	4.80 (−49.85–21.90)	5.80 (−10.70–27.10)	0.76	−0.15
	L1-L2	1.15 (−42.0–13.95)	1.33 (−42.00–10.15)	0.95 (−14.80–13.95)	0.79	−0.05
PWVao (m/s)	L1	8.25 (5.75–14.40)	8.10 (5.80–12.60)	8.30 (5.75–14.40)	0.29	0.35
	S	9.20 (6.90–14.45)	10.15 (7.20–14.45)	9.00 (6.90–13.40)	0.002	1.05
	L2	8.28 (4.40–15.25)	8.43 (5.20–13.15)	8.18 (4.40–15.25)	0.33	0.25
ΔPWVao (m/s)	L1-S	1.15 (−5.40–8.65)	1.60 (−5.40–8.65)	0.90 (−3.95–5.25)	0.01	0.70
	S-L2	−1.08 (−8.60–2.50)	−1.53 (−8.60–0.80)	−0.63 (−5.15–2.50)	0.01	−0.70
	L1-L2	0.08 (−7.40–3.15)	0.13 (−7.40–2.10)	0.05 (−3.90–3.15)	0.42	0.10

All values are presented as the median (range). Abbreviations: Δ—change in value between two test phases; AIxA—aortic augmentation index; AIxB—brachial augmentation index; F—females; HL—Hodges–Lehman; HR—heart rate; L1—supine 1; L2—supine 2; LSL—lay-stand-lay test; M—males; PPao—aortic pulse pressure; PWVao—aortic pulse wave velocity; RR—respiratory rate; S—standing; and SBPao—aortic systolic blood pressure.

**Table 3:** Spearman correlation matrix between anthropometric and psychological variables.

	YCI-SC	YCI-I	YCI-PC	YCI-AVG	LEI	EQ-5D-3L
Age	−0.25 *	−0.32 **	−0.18	−0.29 *	−0.07	−0.03
Weight	0.13	0.02	0.05	0.14	0.04	−0.08
Height	0.02	0.11	−0.13	0.05	−0.07	0.07
BMI	0.10	−0.04	0.07	0.08	0.07	−0.11
Waist circumference	0.08	−0.03	0.08	0.05	0.06	−0.12
Hip circumference	0.03	−0.05	0.07	0.03	0.01	−0.08
WHR	0.02	−0.14	0.04	−0.03	0.00	−0.12
WHtR	0.09	−0.06	0.11	0.04	0.06	−0.14
	Females					
Age	−0.32 *	−0.28	−0.19	−0.35 *	−0.08	−0.05
Weight	0.10	−0.04	0.12	0.10	0.15	−0.10
Height	−0.14	0.04	−0.25	−0.08	−0.05	0.10
BMI	0.11	−0.04	0.15	0.10	0.18	−0.10
Waist circumference	0.04	−0.11	0.17	0.03	0.18	−0.13
Hip circumference	−0.04	−0.11	0.09	−0.02	0.07	−0.05
WHR	0.04	−0.19	0.14	0.01	0.14	−0.17
WHtR	0.08	−0.08	0.21	0.06	0.18	−0.15
	Males					
Age	−0.14	−0.36 *	−0.17	−0.19	0.00	0.01
Weight	−0.01	0.04	0.09	0.10	−0.25	−0.09
Height	0.18	0.17	0.31	0.22	−0.13	0.01
BMI	−0.10	−0.08	−0.03	−0.04	−0.22	−0.10
Waist circumference	−0.04	−0.05	0.08	−0.01	−0.24	−0.14
Hip circumference	0.04	0.02	0.11	0.07	−0.29	−0.03
WHR	−0.17	−0.14	−0.11	−0.19	−0.32	0.01
WHtR	−0.07	−0.14	−0.01	−0.07	−0.24	−0.11

* q < 0.05 and ** q < 0.01. Abbreviations: BMI—body mass index; EQ-D5-3L—health-related quality-of-life questionnaire—quality-of-life score; LEI—Holmes–Rahe Life Events Inventory Score; waist-to-hip ratio; WHR—waist-to-hip ratio; WHtR—waist-to-height ratio; YCI—Young Hypercompensation Inventory; YCI-AVG—YCI average score, YCI-I—YCI individuality, YCI-PC—YCI personal control, YCI-SC—YCI social control, q—Benjamini–Hochberg FDR within outcome.

**Table 4:** FDR-significant multivariable associations between psychological outcomes and anthropometric predictors, stratified by sex, with sex × predictor interaction tests (male estimates included).

Outcome	Predictor	F β_std [95% CI]	q_F	R^2^_F	n_F	M β_std [95% CI]	q_M	R^2^_M	n_M	q_Interaction
YCI-AVG	Age	−0.43 [−0.69, −0.16]	0.011	0.217	80	-				0.499
YCI-SC	Age	−0.41 [−0.68, −0.13]	0.021	0.18	80	-				0.523
YCI-PC	Age	−0.42 [−0.67, −0.16]	0.009	0.263	80	-				0.918
YCI-PC	Waist (cm)	0.70 [0.24, 1.17]	0.009	0.263	80	-				0.607
YCI-PC	Hips (cm)	−0.61 [−1.05, −0.17]	0.012	0.263	80	-				0.918

Standardized OLS per outcome with anthropometric predictors (z) and covariates (smoking and partnership) fit separately in women and men with VIF-based pruning. q_FDR values are Benjamini–Hochberg within outcome and sex. Interaction q-values are derived from pooled single-predictor models (y ~ sex + predictor + sex × predictor + covariates), adjusted within outcome. Where a predictor was pruned from the male multivariable model due to collinearity, the male effect size shown is from a single-predictor, covariate-adjusted standardized OLS (provided for comparability; not used for FDR decisions). Abbreviations: β—regression coefficient, CI—confidence interval, F—females, M—males, n—number of complete cases for that outcome, OLS—ordinary least squares, R^2^—coefficient of determination, YCI—Young Hypercompensation Inventory, YCI-AVG—YCI average score, YCI-PC—YCI personal control, and YCI-SC—YCI social control.

## Data Availability

The data are available from the corresponding author upon reasonable request.

## Supplementary Materials

The following supporting information can be downloaded at: https://www.mdpi.com/article/10.3390/medicina61091682/s1, Table S1. Influence of demographic and lifestyle factors on ANS reactivity–LMM c1 interactions and Δ-models (FDR-controlled), Table S2. Influence of anthropometric factors on ANS reactivity–FDR-significant Δ-models (ANCOVA, HC3), Table S3. Comparison of changes in physiological parameters (delta values) during the LSL test between obese and non-obese participants, Table S4. FDR-significant associations of continuous central adiposity with AIxA, AIxB deltas, Table S5. Top 5 (by FDR q) nominal associations between psychological measures and autonomic reactivity.
