# Supplementary material for: Age, Sex, and Central Adiposity as Determinants of Autonomic Nervous System Reactivity in Adults
Source: Medicina (Kaunas). 2025 Sep 17;61(9):1682. doi: 10.3390/medicina61091682 (PMC12471804; doi:10.3390/medicina61091682)
Supplement: Supplementary file 1 [file medicina-61-01682-s001.zip › medicina-3839225-supplementary.pdf]

medicina

MDPI

Supplementary Tables

Supplementary Table S1. Influence of demographic and lifestyle factors on ANS reactivity – LMM c1 interactions and Δ-models (FDR-controlled).

LMM (c1/c2 interactions)						
**Outcome**	**Delta/Contrast**	**Predictor**	**Effect (β [95% CI])**	**q**	**R^2**	**n**
AIxA	c1	Age	-1.98 [-2.80, -1.16]	0.00		357
AIxA	c1	Smoking	3.58 [1.78, 5.38]	0.00		357
AIxB	c1	Age	-3.64 [-5.38, -1.91]	0.00		356
AIxB	c1	Smoking	7.02 [3.22, 10.82]	0.00		356
PPao	c1	Age	-1.11 [-1.84, -0.38]	0.02		356
PWVao	c1	Age	-0.46 [-0.61, -0.30]	0.00		357
PWVao	c1	Sex M	0.48 [0.17, 0.79]	0.01		357
SBPao	c1	Age	-2.40 [-3.34, -1.46]	0.00		356
SBPao	c1	Smoking	2.68 [0.61, 4.75]	0.04		356
**Δ-model (ANCOVA, HC3)**						
**Outcome**	**Delta/Contrast**	**Predictor**	**Effect (β [95% CI])**	**q**	**R^2**	**n**
AIxA	ΔSL2	Age	6.06 [4.25, 7.88]	0.00	0.39	119
AIxA	ΔSL2	Smoking	-4.32 [-7.70, -0.95]	0.02	0.39	119
AIxA	ΔL1L2	Age	1.20 [0.48, 1.92]	0.00	0.13	119
AIxB	ΔSL2	Age	11.22 [7.33, 15.11]	0.00	0.34	118
AIxB	ΔSL2	Smoking	-8.42 [-15.34, -1.49]	0.03	0.34	118
PPao	ΔSL2	Age	3.24 [1.61, 4.87]	0.00	0.25	118
PWVao	ΔL1S	Sex M	0.86 [0.20, 1.52]	0.04	0.37	119
PWVao	ΔSL2	Age	0.90 [0.67, 1.13]	0.00	0.49	119
RR	ΔL1L2	Sex	-0.77 [-1.29, -0.24]	0.02	0.14	117
SBPao	ΔSL2	Age	4.94 [2.76, 7.11]	0.00	0.28	118

Predictors: sex_M (1=male), smoking (1/0), partnership (1/0), age_z (per 1 SD). Δ-models: baseline-adjusted (ΔL1S|L1; ΔSL2|S; ΔL1L2|L1); HC3 robust SE; FDR within (Outcome×Delta). LMM: orthogonal contrasts c1 (L1→S), c2 (S→L2); random intercept (ID); FDR within (Outcome) for interactions.

Abbreviations: β – regression coefficient, Δ – change in value (delta) between two phases of the test, AIxA – aortic augmentation index, AIxB – brachial augmentation index, ANCOVA — analysis of covariance, CI – confidence interval, FDR — false discovery rate, HC3 — Heteroskedasticity-Consistent (type 3) standard errors, L1 – supine 1, L2 – supine 2, LMM — linear mixed-effects model, M – males, n - for Δ-models: number of complete cases for that outcome/delta; for LMM: total observations contributing (participants × phases) after listwise deletion, PPao – aortic pulse pressure, PWVao – aortic pulse wave velocity, q – Benjamini–Hochberg FDR within outcome, R^2 – coefficient of determination, RR – respiratory rate, S – stand, SBPao – aortic systolic blood pressure.

Supplementary Table S2. Influence of anthropometric factors on ANS reactivity – FDR-significant Δ-models (ANCOVA, HC3)

Outcome	Delta	Predictor	Effect (β [95% CI])	q	R^2	n
HR	ΔSL2	Hips_cm	-5.16 [-8.14, -2.19]	0.00	0.42	119
AIxA	ΔL1S	Waist_cm	2.26 [0.78, 3.74]	0.00	0.32	118
AIxA	ΔSL2	Waist_cm	-1.97 [-3.36, -0.59]	0.02	0.44	118
AIxA	ΔSL2	Hips_cm	-8.60 [-11.15, -6.04]	0.00	0.39	119
AIxA	ΔL1L2	Hips_cm	-2.07 [-3.50, -0.64]	0.03	0.13	119
AIxA	ΔL1S	BMI	2.04 [0.90, 3.17]	0.00	0.35	117
AIxA	ΔSL2	BMI	-1.58 [-2.79, -0.37]	0.02	0.41	117

AIxA	ΔL1S	WHR	2.45 [1.05, 3.85]	0.00	0.36	117
AIxA	ΔSL2	WHR	-2.01 [-3.64, -0.38]	0.02	0.41	117
AIxA	ΔL1S	WHtR	2.15 [0.82, 3.49]	0.00	0.33	118
AIxA	ΔSL2	WHtR	-1.70 [-2.96, -0.43]	0.02	0.44	118
AIxB	ΔL1S	Waist_cm	4.46 [1.53, 7.38]	0.00	0.32	117
AIxB	ΔSL2	Waist_cm	-4.59 [-7.54, -1.64]	0.01	0.39	117
AIxB	ΔL1S	Hips_cm	10.44 [5.47, 15.40]	0.00	0.29	118
AIxB	ΔSL2	Hips_cm	-6.91 [-11.50, -2.31]	0.01	0.34	118
AIxB	ΔL1S	BMI	4.05 [1.80, 6.30]	0.00	0.35	116
AIxB	ΔSL2	BMI	-3.60 [-6.15, -1.05]	0.01	0.36	116
AIxB	ΔL1S	WHR	4.85 [2.09, 7.61]	0.00	0.36	116
AIxB	ΔSL2	WHR	-4.13 [-7.56, -0.69]	0.03	0.36	116
AIxB	ΔL1S	WHtR	4.25 [1.61, 6.89]	0.00	0.33	117
AIxB	ΔSL2	WHtR	-3.90 [-6.59, -1.20]	0.01	0.39	117
SBPao	ΔL1S	Waist_cm	2.48 [0.84, 4.13]	0.01	0.34	118
SBPao	ΔSL2	Hips_cm	-12.83 [-21.00, -4.65]	0.01	0.28	118
SBPao	ΔL1S	WHtR	2.27 [0.72, 3.82]	0.01	0.34	118
PPao	ΔL1S	Waist_cm	1.85 [0.65, 3.05]	0.01	0.34	118
PPao	ΔL1S	Hips_cm	-4.72 [-8.00, -1.45]	0.01	0.30	119
PPao	ΔSL2	Hips_cm	-9.03 [-11.83, -6.23]	0.00	0.25	118
PPao	ΔL1L2	Hips_cm	-3.82 [-5.97, -1.68]	0.00	0.13	118
PPao	ΔL1S	BMI	1.31 [0.22, 2.41]	0.03	0.33	117
PPao	ΔL1S	WHR	1.79 [0.21, 3.38]	0.04	0.35	117
PPao	ΔL1S	WHtR	1.72 [0.64, 1.79]	0.01	0.35	118
PWVao	ΔL1S	Hips_cm	-1.82 [-2.95, -0.70]	0.01	0.38	119
PWVao	ΔSL2	Hips_cm	-1.40 [-2.01, -0.79]	0.00	0.49	119

Anthropometric predictors (z-scored): Height_cm, Weight_kg, Waist_cm, Hips_cm, BMI, WHR, WHtR. Δ-models: HC3 robust SE; baselines ΔL1S∣L1, ΔSL2∣S, ΔL1L2∣L1; FDR within (Outcome×Delta) across anthropometric terms. β is per 1 SD in predictor; sex_M = men vs. women; smoking/partner binary; age_z per 1 SD.

Abbreviations: β – regression coefficient, Δ – change in value (delta) between two phases of the test, AIxA – aortic augmentation index, AIxB – brachial augmentation index, ANCOVA — analysis of covariance, CI – confidence interval, FDR — false discovery rate, HC3 — Heteroskedasticity-Consistent (type 3) standard errors, L1 – supine 1, L2 – supine 2, n - for Δ-models: number of complete cases for that outcome/delta, PPao – aortic pulse pressure, PWVao – aortic pulse wave velocity, R^2 – coefficient of determination, q – Benjamini–Hochberg FDR within outcome, RR – respiratory rate, S – stand, SBPao – aortic systolic blood pressure.

Supplementary Table S3. Comparison of changes in physiological parameters (delta values) during the LSL test between obese and non-obese participants.

Delta Parameter	Average (Non-Obese)	SD (Non-Obese)	Average (Obese)	SD (Obese)	t-value	p
ΔRR_L1S	-0.19	1.66	-0.69	2.12	1.34	0.19
ΔRR_SL2	0.39	1.62	0.78	2.23	-0.99	0.32
ΔRR_L1L2	0.21	1.28	0.09	1.20	0.49	0.62
ΔHR_L1S	17.06	7.51	14.85	6.70	1.68	0.10
ΔHR_SL2	-18.10	8.29	-16.28	7.20	-1.27	0.21
ΔHR_L1L2	-1.04	2.53	-1.43	3.06	0.74	0.46
ΔAIxB_L1S	-14.48	17.58	-11.15	17.85	-0.99	0.32
ΔAIxB_SL2	19.73	19.23	15.63	18.07	1.18	0.24
ΔAIxB_L1L2	5.25	10.76	4.48	5.63	0.52	0.61
ΔSBPao_L1S	2.96	10.29	2.91	10.04	0.03	0.98

ΔSBPao_SL2	-4.29	16.62	-1.60	11.02	-1.06	0.29
ΔSBPao_L1L2	-1.33	14.65	1.31	5.26	-1.41	0.16
ΔPPao_L1S	-5.48	8.09	-4.58	6.10	-0.69	0.49
ΔPPao_SL2	5.51	10.05	6.07	6.46	-0.37	0.71
ΔPPao_L1L2	0.03	6.36	1.49	4.97	-1.40	0.16
ΔAIxA_L1S	-7.34	8.89	-5.62	9.08	-1.01	0.32
ΔAIxA_SL2	9.42	9.23	7.92	9.15	0.87	0.39
ΔAIxA_L1L2	2.08	3.48	2.29	2.85	-0.36	0.72
ΔPWVao_L1S	1.21	1.90	1.38	1.59	-0.54	0.59
ΔPWVao_SL2	-1.35	1.55	-1.05	1.70	-0.97	0.33
ΔPWVao_L1L2	-0.14	1.24	0.33	0.75	-2.60	**0.01**

Abbreviations: Δ – change in value (delta) between two phases of the test, AIxA – aortic augmentation index, AIxB – brachial augmentation index, HR – heart rate, L1 – supine 1, L2 – supine 2, PPao – aortic pulse pressure, PWVao – aortic pulse wave velocity, RR – respiratory rate, S – stand, SBPao aortic systolic blood pressure, SD – standard deviation.

Supplementary Table S4. FDR-significant associations of continuous central adiposity with AIxA, AIxB deltas.

Outcome	Delta	Predictor	Effect (β [95% CI])	q	R^2	n
AIxA	ΔL1S	WHR	2.62 [0.86, 4.38]	0.02	0.25	117
AIxA	ΔL1S	WHtR	2.39 [0.68, 4.11]	0.02	0.23	118
AIxA	ΔL1S	Waist_cm	2.61 [0.68, 4.53]	0.02	0.23	118
AIxA	ΔSL2	WHR	-2.82 [-4.65, -1.00]	0.02	0.23	117
AIxA	ΔSL2	WHtR	-2.38 [-4.16, -0.59]	0.02	0.22	118
AIxA	ΔSL2	Waist_cm	-2.58 [-4.59, -0.57]	0.02	0.21	118
AIxB	ΔL1S	WHR	5.18 [1.70, 8.66]	0.01	0.25	116
AIxB	ΔL1S	WHtR	4.74 [1.34, 8.14]	0.01	0.24	117
AIxB	ΔL1S	Waist_cm	5.15 [1.33, 8.97]	0.01	0.23	117
AIxB	ΔSL2	WHR	-5.74 [-9.59, -1.89]	0.01	0.19	116
AIxB	ΔSL2	WHtR	-5.17 [-8.93, -1.42]	0.01	0.18	117
AIxB	ΔSL2	Waist_cm	-5.73 [-9.94, -1.52]	0.01	0.18	117

Models adjusted for age (z), sex, smoking, and partnership.
Abbreviations: β – regression coefficient, Δ – change in value (delta) between two phases of the test, AIxA – aortic augmentation index, AIxB – brachial augmentation index, CI – confidence interval, FDR — false discovery rate, L1 – supine 1, L2 – supine 2, q – Benjamini–Hochberg FDR within outcome, R2 – determination coefficient, S – stand, WHR – waist-to-hip ratio, WHtR – waist-to-height ratio.

Supplementary Table 5. Top 5 (by FDR q) nominal associations between psychological measures and autonomic reactivity

Outcome	Delta	Predictor	Effect (β [95% CI])	q	R^2	n
AIxA	ΔL1L2	YCI-PC	-0.24 [-0.42, -0.05]	0.23	0.06	118
AIxA	ΔSL2	YCI-PC	-0.17 [-0.35, 0.00]	0.48	0.19	118
AIxA	ΔSL2	YCI-SC	-0.07 [-0.25, 0.10]	0.86	0.17	119
AIxA	ΔL1L2	YCI-AVG	-0.11 [-0.31, 0.08]	0.86	0.02	119
AIxA	ΔSL2	YCI-AVG	-0.08 [-0.26, 0.11]	0.86	0.17	119
AIxB	ΔL1L2	YCI-PC	-0.16 [-0.29, -0.03]	0.34	0.06	118
AIxB	ΔSL2	YCI-PC	-0.16 [-0.33, 0.01]	0.58	0.18	117
AIxB	ΔL1L2	YCI-AVG	-0.11 [-0.31, 0.08]	0.89	0.03	119
AIxB	ΔSL2	YCI-AVG	-0.09 [-0.27, 0.10]	0.89	0.13	118
AIxB	ΔSL2	LEI	0.11 [-0.08, 0.29]	0.89	0.14	115
PPao	ΔSL2	LEI	0.20 [0.01, 0.39]	0.66	0.11	114

PPao	ΔL1L2	LEI	0.12 [-0.07, 0.31]	0.82	0.02	114
PPao	ΔSL2	YCI-I	0.10 [-0.09, 0.29]	0.82	0.07	118
PPao	ΔL1L2	YCI-I	0.10 [-0.10, 0.30]	0.82	0.01	118
PPao	ΔL1S	EQ-5D-3L	0.11 [-0.07, 0.29]	0.82	0.08	119
PWVao	ΔSL2	YCI-SC	0.16 [-0.01, 0.33]	0.76	0.24	119
PWVao	ΔL1S	YCI-SC	-0.16 [-0.33, 0.02]	0.76	0.17	119
PWVao	ΔSL2	YCI-I	0.04 [-0.13, 0.21]	0.82	0.22	119
PWVao	ΔSL2	YCI-AVG	0.13 [-0.04, 0.30]	0.82	0.23	119
PWVao	ΔSL2	LEI	-0.03 [-0.20, 0.14]	0.82	0.21	115
SBPao	ΔSL2	LEI	0.17 [-0.00, 0.35]	0.51	0.19	114
SBPao	ΔL1S	LEI	-0.17 [-0.35, 0.01]	0.51	0.19	115
SBPao	ΔL1L2	EQ-5D-3L	0.01 [-0.18, 0.20]	0.99	0.01	118
SBPao	ΔSL2	YCI-I	0.04 [-0.14, 0.22]	0.99	0.16	118
SBPao	ΔSL2	YCI-AVG	0.04 [-0.14, 0.22]	0.99	0.16	118
HR	ΔL1L2	EQ-5D-3L	0.14 [-0.04, 0.33]	0.54	0.06	119
HR	ΔSL2	YCI-I	-0.17 [-0.37, 0.02]	0.54	0.05	119
HR	ΔL1L2	YCI-SC	-0.16 [-0.35, 0.03]	0.54	0.06	119
HR	ΔL1S	YCI-I	0.16 [-0.04, 0.35]	0.54	0.04	119
HR	ΔSL2	YCI-AVG	-0.07 [-0.27, 0.12]	0.73	0.03	119

Standardized OLS models (z outcome, z predictor) adjusted for age, sex, smoking, and partnership. FDR (Benjamini–Hochberg) applied within each physiological outcome; table shows the top-5 rows by smallest q for transparency—no association reached q<0.05.

Abbreviations: β – standardized regression coefficient, Δ – change in value (delta) between two phases of the test, AIxA – aortic augmentation index, AIxB – brachial augmentation index, CI – confidence interval, FDR — false discovery rate, L1 – supine 1, L2 – supine 2, n - number of complete cases for that outcome, PPao – aortic pulse pressure, PWVao – aortic pulse wave velocity, q – Benjamini–Hochberg FDR within outcome, R^2 – coefficient of determination, RR – respiratory rate, S – stand, SBPao – aortic systolic blood pressure.
